# Volume-Based Quantitative Measurement of [^18^F]AlF-NOTA-FAPI-04 PET/CT Uptake Reflects the Disease Activity of IgG4-Related Disease

**DOI:** 10.1007/s11307-024-01928-8

**Published:** 2024-07-30

**Authors:** Liyan Wan, Chuanyin Sun, Junyu Liang, Jin Lin, Zhi Chen

**Affiliations:** 1 Department of Rheumatology, The First Affiliated Hospital, College of Medicine, Zhejiang University, 79 Qingchun Road, Hangzhou, 310003 Zhejiang Province China; 2 Department of Infectious Diseases, State Key Laboratory for Diagnosis and Treatment of Infectious Diseases, National Clinical Research Center for Infectious Diseases, Collaborative Innovation Center for Diagnosis and Treatment of Infectious Diseases, The First Affiliated Hospital, College of Medicine, Zhejiang University, 79 Qingchun Road, Hangzhou, 310003 Zhejiang Province China

**Keywords:** Positron emission tomography/computed tomography, IgG4-related disease, Disease activity

## Abstract

**Background:**

To investigate the potential utility of quantitative parameters obtained by ^18^F-fibroblast activation protein inhibitor positron emission tomography/computed tomography ([^18^F]AlF-NOTA-FAPI-04 PET/CT) in the assessment of organ involvement and disease activity in IgG4-related disease (IgG4-RD).

**Methods:**

This study enrolled patients who underwent [^18^F]AlF-NOTA-FAPI-04 PET/CT scans at the Department of Rheumatology, The First Affiliated Hospital, Zhejiang University School of Medicine from August 2021 to August 2022. The PET/CT images of the included patients were re-evaluated by PET center technicians, and the maximal standardized uptake value (SUV_max_), metabolic lesion volume (MLV), and total lesion FAPI (TL-FAPI) were used to evaluate the involved organs and tissues that abnormally accumulated [^18^F]AlF-NOTA-FAPI-04. The clinical and laboratory data of patients are also systematically collected and analyzed.

**Results:**

Among the patients included in this study, 12 patients met the IgG4-RD classification criteria established by the American College of Rheumatology in 2019. Among them, 8 were males and 4 were females, with an average age of 59.3 ± 11.5 years. 50% of IgG4-RD patients were found with more organ involvement on PET/CT than physical examination, ultrasonography, and computed tomography. IgG4 levels (Rho = 0.594, *p* = 0.042) and IgG4-RI (Rho = 0.647, *p* = 0.023) were significantly positively correlated with TL-FAPI. After linear regression analysis, only TL-FAPI showed a predictive value of RI (*R*^2^ = 0.356, B = 0.008, *p* = 0.041).

**Conclusions:**

[^18^F]AlF-NOTA-FAPI-04 PET/CT is a useful tool for identifying asymptomatic organ involvement and assessing disease activity. The TL-FAPI as an indicator was positively correlated with IgG4-RD disease activity.

## Introduction

Immunoglobulin G4-related disease (IgG4-RD) is an immune-mediated disease characterized by the infiltration of IgG4-positive plasma cells and fibrosis at nearly any anatomic site [1]. Typical organs known to be affected include salivary glands, pancreas, liver, biliary system, retroperitoneum, kidneys, the orbits and lacrimal glands, which makes its clinical symptoms heterogeneous. The laboratory profile of IgG4-RD includes high blood levels of IgG4, IgG, and IgE [2]. Organs involved may demonstrate enlargement on radiological modalities [3]. Diagnosis of IgG4-RD is based on a combination of clinical, serological, radiological, and pathological findings after an exclusion of malignancy, other immune-mediated diseases, or infection [1].

Although conventional imaging modalities help the careful evaluation of a local lesion, they provide limited value in whole-body evaluation, especially in identifying the asymptomatic lesions. ^18^F-fluorodesoxyglucose positron emission tomography/computed tomography (^18^F-FDG PET/CT), which reflects both the metabolic activity and the anatomical structure of multiorgan lesions in one scan, is widely used in the diagnosis, staging and treatment response of malignancies [4] and inflammatory diseases [5–7]. Several studies have reported that ^18^F-FDG PET/CT is a valuable tool to evaluate patients with IgG4-RD for the detection of involved-organs [8, 9], guiding tissue biopsy [10] and monitoring response to therapy[11, 12]. Also, ^18^F-FDG PET/CT semiquantitative parameters correlated with circulating plasmablasts rather than processes related to fibroblast activation and extracellular matrix deposition as shown in previous study [13].

Fibroblast activation protein (FAP) is highly expressed in activated fibroblasts at tumor microenvironment and wound healing/inflammatory sites [14]. Due to the very low expression of FAP in normal tissue and a rapid elimination from the circulation, fibroblast activation protein inhibitors (FAPI) show a higher target to background ratio and a better sensitivity than FDG [15]. ^68^Ga-labeled FAP probes have recently been widely used for the detection of IgG4-RD related lesions. In 2020, Schmidkonz et al. showed that ^68^Ga-FAPI tracer uptake is associated with fibrotic but not inflammatory lesions of IgG4-RD [16], which introduced FAPI as a promising tracer in the detection of IgG4-RD. In 2021, Luo et al. revealed that ^68^Ga-FAPI had a higher rate of detecting involvement of the pancreas, bile duct/liver, and salivary gland [17]. However, ^68^Ga-FAPI PET/CT imaging suffers the disadvantage of a radionuclide supply. The longer half-life of ^18^F and the availability of ^18^F from cyclotrons that are owned by many medical centers offer a favorable property of ^18^F. Also, the lower positron energy of ^18^F (0.65 MeV) than ^68^Ga (1.90 MeV) may lead to less scattering of high-energy positrons and higher spatial resolution [18]. However, in previous study, ^68^Ga-FAPI-04 showed lower accumulation of radioactivity in the parotid gland, submandibular gland, thyroid, biliary tract, and pancreas than ^18^F-FAPI-42 [19]. It is not precisely clear whether ^18^F performs as well as ^68^Ga in the detection of IgG4-RD.

Here, we quantified [^18^F]AlF-NOTA-FAPI-04 uptake in patients with IgG4-RD using the maximum and mean standardized uptake value (SUV_max_ and SUV_mean_), metabolic tumor volume (MTV) and total lesion FAPI (TL-FAPI), in pursuit of identifying markers associated with disease burden as well.

## Material and Methods

### Patient Recruitment

From August 2021 to August 2022, 21 patients suspected with IgG4-RD in the Department of Rheumatology of the First Affiliated Hospital, Zhejiang University School of Medicine were included in the study. Among them, 12 patients fulfilled the 2019 American College of Rheumatology/European League Against Rheumatism (2019 ACR/EULAR) classification criteria for IgG4-RD [1]. Clinical manifestations and laboratory data were obtained from the current hospitalization. Clinical phenotypes of IgG4-RD were divided into four groups according to a previous report: head and neck–limited group, Mikulicz and systemic group, retroperitoneum and aorta group and pancreato-hepatobiliary group [20]. The IgG4-RD responder index (IgG4-RI) was calculated according to Wallace et al. [21] at the time of PET/CT scan. Findings of conventional radiological imaging including ultrasonography (US), computer tomography (CT), and magnetic resonance imaging (MRI) were also recorded in comparison to PET/CT results. In addition, 9 patients were ultimately diagnosed with other autoimmune diseases (2 with antineutrophilic cytoplasmic antibody-associated vasculitis, 2 with histiocytic necrotic lymphadenitis, 2 with systemic lupus erythematosus, 2 with hyperglobulinemia, 1 with rheumatoid arthritis), recruiting as the control group for this study. Written informed consent was waived due to the retrospective nature of this study. The study was approved by the Ethics Committee of the first affiliated hospital, Zhejiang University School of Medicine.

### PET/CT Scanning

[^18^F]AlF-NOTA-FAPI-04 [22] PET/CT scanning was performed using the Siemens Biograph vision 600 system. Patients received an intravenous injection of 3.7 MBq of [^18^F]AlF-NOTA-FAPI-04 per kilogram of body weight 50–70 min before scanning from the skull base to the mid-thigh. PET images were acquired for 2 min per bed position using a matrix size of 440 × 440, 5 subsets, 10 iterations, and 4.0 mm full-width half-maximum post-filtering. CT images were acquired using a tube voltage of 120 kV, CARE Dose 4D technology auto modulates the tube current, a section thickness of 3 mm and the pitch 1.0. The reconstruction of PET images was based on TrueX + TOF with photon attenuation correction using CT data.

### PET/CT Image Interpretation

Integrated PET and CT images were independently interpreted by two nuclear medicine physicians on the Syngo.via work station. Hyper-uptake lesions were identified based on a visual comparison of [^18^F]AlF-NOTA-FAPI-04 uptake between the background organ and target site. Volumes of interest (VOI) were drawn around lesions on transaxial slices and automatically adapted to a 3-dimensional volume of interest at a 41% isocontour of the SUV_max_. The tracer accumulations were quantified by the SUV_max_ and SUV_mean_ within the VOI. Evaluation of spleen and liver was conducted with a 2-cm diameter sphere placed inside the organ parenchyma. The SUV_max_ of bone marrow was obtained from lumbar vertebrae 1–5. In this study, the MTV was renamed as the metabolic lesion volume (MLV) because IgG4-RD affected lesions were non-neoplastic. The MLV was automatically delineated using a threshold of 41% of the SUV_max_, then the TL-FAPI was calculated as the MLV multiplied by the SUV_mean_. Both the MLV and TL-FAPI were measured on each hyper-uptake lesion. The total MLV (MLV_total_), which summed MTVs, and the TL-FAPI, which summed TL-FAPIs, in all hyper-uptake lesions were measured as the quantitative parameters of whole-body disease burden.

### Statistical Analysis

Statistical analysis was performed using SPSS (version 23, IBM Corporation) and R (version 3.6.2) software packages. Chi-Square and Fisher’s exact teats were used to compare categorical variables. The Mann–Whitney rank sum test was used to compare continuous variables with non-normal distribution after the Kolmogorov–Smirnov test. The Wilcoxon signed-rank test was used to compare paired continuous variables with non-normal distribution. Spearman’s correlation correlations were analyzed to examine the relationship between PET/CT parameters and laboratory indices with a Bonferroni correction. Binary logistic regression and Firth logistic regression [23] were used to evaluate the contribution of different clinical risk factors to disease severity. All the variables were included in the regression mode. The variables with *p* < 0.1 in univariate analysis were entered in multivariate analysis. Multicollinearity was excluded from the final model. Receiver operating characteristic curves (ROCs) were performed to determine the diagnostic performance of the PET/CT parameters. The optimum cut-off value was defined based on the maximum Youden index. The diagnostic accuracy of each parameter was reported together with its 95% confidence interval (CI). Statistical significance was defined as *p* < 0.05.

## Results

### Clinical Information of Recruited Patients

The baseline characteristics of recruited patients were presented in Table 1. This study included 12 patients (8 male and 4 female) with IgG4-RD with the mean age 59. As for control group, there were nine patients (8 female and 1 male) with the mean age 52. The clinical, pathologic, and biologic characteristics of patients with IgG4-RD were presented in Table 2. Clinical phenotypes of IgG4-RD were Mikulicz and systemic (9 patients), retroperitoneum and aorta (3 patients). The mean IgG4-RD responder index (RI) was 12.9. Nine patients were treatment naïve at the time of PET/CT scanning, while 3 patients with recurrent or persistent manifestations during the treatment including glucocorticoids and disease modifying anti-rheumatic drugs (DMARDs). Among IgG4-RD group, six patients did not receive tissue biopsy. One patient received core needle biopsy of retroperitoneum, two patients received core needle biopsy of prostate after PET/CT scanning, while one patient receive lymphadenectomy, two patients underwent excision of submandibular gland before PET/CT scanning. All of the histopathology of biopsy tissue helped to clear the diagnosis.

**Table 1:** The baseline characteristics of recruited patients

	IgG4-RD (*n* = 12)	Disease control (*n* = 9)	*p*
Age	59.33 ± 11.54	52.44 ± 20.77	0.344
Male	8	1	0.024*
Disease			
IgG4-RD	12	0	
ANCA-associated vasculitis	0	2	
Histiocytic necrotic lymphadenitis	0	2	
systemic lupus erythematosus	0	2	
Hyperglobulinemia	0	2	
Rheumatoid arthritis	0	1	
Laboratory findings			
WBC	6.06(4.60, 7.55)	5.67(4.25, 10.88)	0.569
Hb	127(119, 130)	96(84, 124)	0.017*
PLT	186(173, 288)	223(155, 312)	0.849
Eosinophils count	0.21(0.12, 0.22)	0.09(0.07, 0.22)	0.304
CRP	3.0(3.0, 17.2)	36.5(3.8, 52.1)	0.051
ESR	44(20, 51)	58(19, 88)	0.347
IL-6	4.08(2.06, 6.41)	14.35(4.60, 76.2)	0.072
IgG	22.69(15.98, 26.86)	14.66(10.36, 26.88)	0.160
IgG4	5.43(1.48, 26.95)	1.56(0.57, 2.24)	0.037
C3	95(84, 115)	101(91, 128)	0.425
C4	16(10, 35)	23(15, 30)	0.824
PET/CT parameters			
SUV_max_ of bone marrow	1.57 ± 0.34	1.69 ± 0.49	0.538
SUV_mean_ of bone marrow	0.61 ± 0.25	0.57 ± 0.18	0.672
SUV_max_ of liver	2.58 ± 1.03	1.96 ± 0.62	0.129
SUV_mean_ of liver	0.95 ± 0.30	0.80 ± 0.26	0.250
SUV_max_ of spleen	1.91 ± 0.45	1.70 ± 0.60	0.376
SUV_mean_ of spleen	1.14 ± 0.35	1.00 ± 0.50	0.457

*IgG4-RD* IgG4-related disease, *ANCA* Antineutrophilic cytoplasmic antibody, *WBC* white blood cell, *Hb* Hemoglobin, *PLT* platelet, *ESR* erythrocyte sedimentation rate, *CRP* c-reactive protein, *IL-6* interleukin-6, *SUV* standardized uptake value

**Table 2:** Summary of characteristics of patients with IgG4-RD

Patient no	Age (year)/Gender	Organ involvement found before PET/CT	Additional involvement detected by PET/CT	Serum IgG4 (g/L)	hyper-uptake site	IgG4-RI	SUV_max_
1	57/M	Retroperitoneum#, lymphadenopathy	Salivary glands, aorta	4.34	\	9	10.38
2	54/F	Pancreas#, bile duct, lacrimal glands, salivary glands, kidney, lymphadenopathy, aorta	\	20.2	Lymph node	29	10.33
3	41/M	Retroperitoneum#	\	0.727	\	13	5.33
4	61/M	Salivary glands, kidney, lymphadenopathy	Pancreas#, lacrimal glands	31.7	Submandibular gland	13	17.72
5	64/M	Pancreas#, salivary glands, lymphadenopathy	Kidney	56.6	Submandibular gland	11	33.16
6	61/M	Retroperitoneum#	\	1.06	Retroperitoneum	12	12.88
7	70/M	Meninges#, prostate, pancreas, pituitary gland	\	0.245	Prostate	10	18.05
8	54/F	Pancreas#, salivary glands	\	10.6	\	7	30.19
9	61/F	Pancreas#	Salivary glands	2.75	\	4	13.02
10	87/M	Retroperitoneum	Pancreas#	3.56	\	11	26.57
11	48/F	Retroperitoneum#, lacrimal glands, salivary glands, pancreas, lymphadenopathy	\	6.53	\	12	8.33
12	54/M	Pancreas#, retroperitoneum, lacrimal glands, salivary glands, lymphadenopathy, prostate, thyroid	Lungs and pleura	29.2	Prostate	24	16.64

^#^Indicates the organ corresponding to the highest glucose uptake use to calculate

### Characterization of Abnormal [^18^F]AlF-NOTA-FAPI-04 Accumulation in Patients with IgG4-RD

**Fig. 1:**
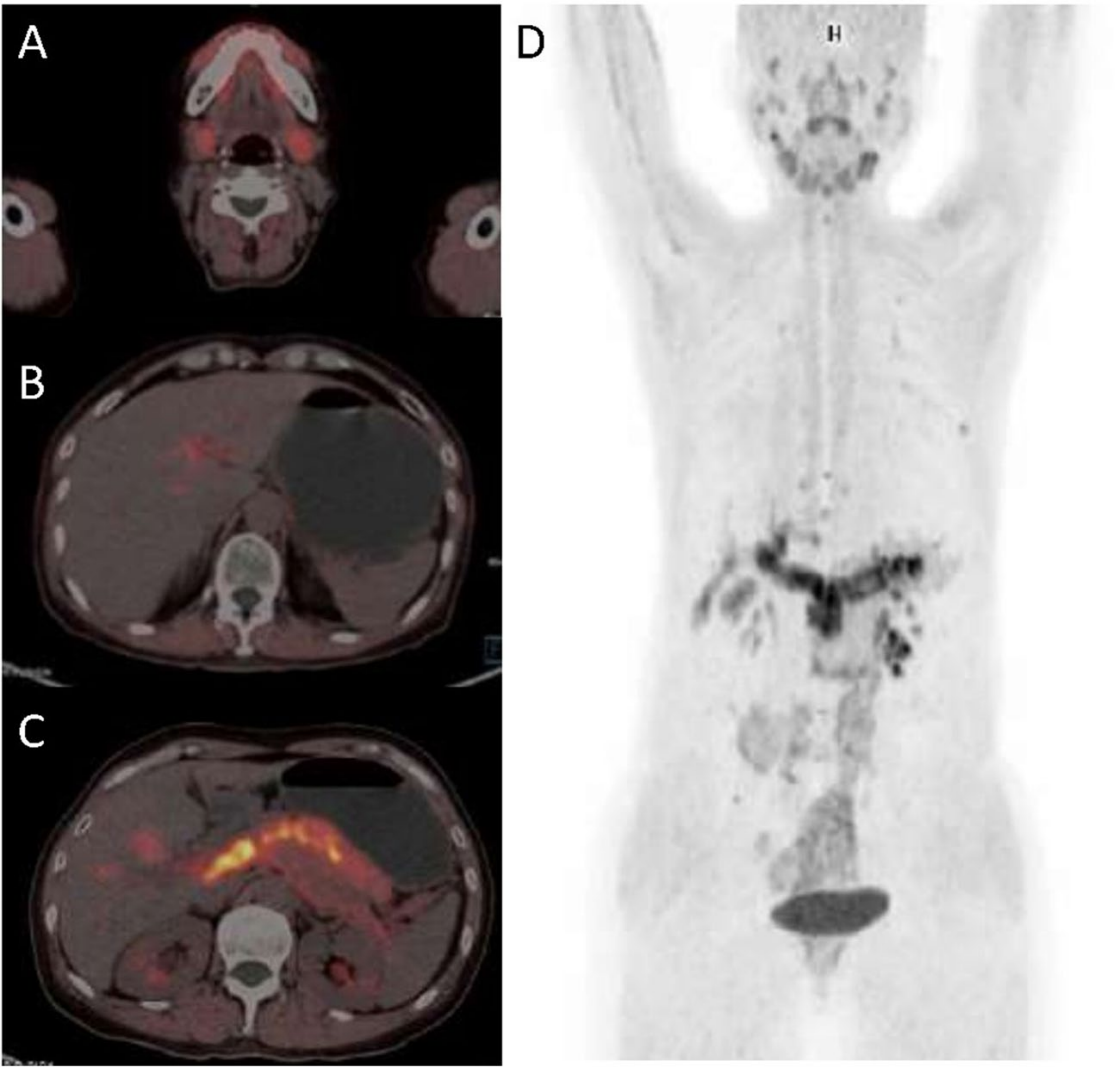
Hybrid PET/CT images of submandibular gland (**A**), bile duct (**B**), pancreas (**C**) and whole-body PET image (**D**), and from a 54-year-old female patient with IgG4-related disease. [^18^F]AlF-NOTA-FAPI-04 PET/CT shows increased [^18^F]AlF-NOTA-FAPI-04 uptake by pancreas (SUV_max_, 10.33), right lacrimal gland (SUV_max_, 6.04), left lacrimal gland (SUV_max_, 5.33), right submandibular gland (SUV_max_, 7.14), and left submandibular gland (SUV_max_, 8.58). SUV, standardized uptake value

In patients with IgG4-RD, disease involvements were observed at more than 14 different known anatomic sites of disease, including lymph nodes, salivary glands, pancreas, retroperitoneal fibrosis, meninges, lung, liver, breast, and lacrimal glands (Table 2). Abnormal [^18^F]AlF-NOTA-FAPI-04 uptake was noticed for all patients with a mean 3.8 organs involved (range 1–8). Involved organs were pancreas (*n* = 9), salivary glands (*n* = 8, parotid and/or submandibular), retroperitoneal fibrosis (*n* = 7), lacrimal glands (*n* = 4), kidneys (*n* = 3), prostate (*n* = 2), aorta and/or other arteries (*n* = 2), lung and pleura, meninges, and thyroid (*n* = 1 each). The mean ± SD SUV_max_ was 16.88 ± 2.55. The highest SUV_max_ values were observed with retroperitoneum, pancreas and meninges involvement. Furthermore, compared with conventional radiologic imaging, FAPI-PET/CT additionally detected 8/45 hyper-uptake organ involvements in 6 of 12 patients, including pancreas (*n* = 2), salivary gland (*n* = 2), aorta, lacrimal gland, kidney, and pleura (*n* = 1 each). In contrast, peripheral lymphadenopathy was observed in 5 patients without abnormal FAPI uptake, while a pathologic analysis of a hyper-uptake lymph node in patient No.2 confirmed typical IgG4-RD histology. Representative PET/CT images of abnormal hyper-uptake distributions in one patient with IgG4-RD were depicted in Fig. 1.

### Correlations Between Clinical Parameters and PET/CT Findings

Among IgG4-RD group, the correlations between disease related biomarkers and PET/CT parameters are summarized in Table 3. The SUV_max_ of bone marrow positively correlated with ESR (Rho = 0.718, *p* = 0.019). SUV_max_ of main involved organ was also significantly positively correlated with ESR (Rho = 0.644, *p* = 0.044). The TL-FAPI displayed a positive correlation with the levels of ESR (Rho = 0.632, *p* = 0.049), IgG4 (Rho = 0.594, *p* = 0.042), and IgG4-RI (Rho = 0.647, *p* = 0.023). Also, the MLV_total_ showed a significantly correlation with IgG4-RI (Rho = 0.682, *p* = 0.015). After linear regression analysis, only TL-FAPI showed a predictive value of RI (R^2^ = 0.356, B = 0.008, *p* = 0.041).

**Table 3:** Correlations between biomarkers and PET/CT parameters in IgG4-RD group

	SUV_max_ of bone marrow		SUV_max_ of main involved organ		TL-FAPI		MLV	
	Rho	*p*	Rho	*p*	Rho	*p*	Rho	*p*
eosinophils	-0.265	0.431	-0.306	0.360	0.420	0.198	0.393	0.232
CRP	0.615	0.059	0.341	0.334	-0.266	0.457	-0.218	0.544
ESR	0.718^*^	0.019	0.644*	0.044	0.632^*^	0.049	0.522	0.122
IgG4	0.126	0.697	0.336	0.286	0.594^*^	0.042	0.462	0.131
IgG4/IgG	-0.127	0.709	0.209	0.537	0.455	0.160	0.291	0.385
IgG4-RI	0.042	0.896	-0.415	0.180	0.647^*^	0.023	0.682^*^	0.015

*CRP* c-reactive protein, *ESR* erythrocyte sedimentation rate, *SUV* standardized uptake value, *TLG* total lesion glycolysis, *MLV* metabolic lesion volume

### PET/CT and Diagnosis of IgG4‑RD

Compared with the disease control group, we found that serum IgG4 concentration was significantly higher in patients with IgG4-RD (5.43 g/L (1.48, 26.95) vs 1.56 g/L (0.57, 2.24), *p* = 0.037). The C-reactive protein in the disease control group was higher than that in the IgG4-RD group with no statistical significance (36.5 (3.8, 52.1) vs 3.0 (3.0, 17.2), *p* = 0.051). Unfortunately, there was no difference between SUV of bone marrow, liver and spleen in the IgG4-RD group and control group. Multivariate logistic analysis was performed to test if the parameters of PET/CT help to diagnose IgG4-RD. Unfortunately, after logistical regression analysis, only hemoglobin associated with the diagnosis of IgG4-related disease, positively (*p* = 0.034, OR = 1.087).

## Discussion

FAPI PET/CT which traced the activated fibroblasts is a useful tool in cancer imaging. Some studies have applied FAPI PET/CT to the evaluation of IgG4-RD, a fibrotic disease. The potential correlations between the PET/CT findings and disease assessments are worth to be investigated. Consistent with ^68^Ga FAPI probes [17, 24–26], [^18^F]AlF-NOTA-FAPI-04 also showed great significance in PET/CT imaging of IgG4-RD patients. The swollen lymph nodes detected by ultrasound and CT were negative in [^18^F]AlF-NOTA-FAPI-04 PET. Further, we demonstrated that TL-FAPI, a summation of tracer uptake in each lesion, correlated significantly and positively with ESR, IgG4, and IgG4-RI. Our study suggests that PET/CT could act as a detective tool to evaluate the disease involvement and assess the disease activity in patients with IgG4-RD.

IgG4-RD group and control group showed comparable level of SUV of bone marrow, liver and spleen, lacking the predictive value in the diagnosis of IgG4-RD. However, the high FAPI uptake accumulated in the organs involved, especially in the pancreas, lacrimal gland and salivary gland, might help to identify the disease pattern and choose biopsy site. Also, FAPI PET/CT help to find additional involved organs and tissues, especially the lacrimal gland, salivary gland and pancreas. Traditional ultrasound, CT, MRI are sensitive to masses or enlargement of pancreas. Luo et al. revealed that FAPI PET/CT additionally detected 8 involved pancreases comparing with FDG PET/CT [17]. Shuo et al. [27] and Zhang et al. [25] also reported cases as FAPI PET detecting the entire pancreas involvements, which were not detected by other scanning. In our study, FAPI PET/CT showed diffuse uptake in pancreas without morphological abnormality, which revealed that FAPI PET/CT detected the accumulation of immune cells and extracellular matrix proteins prior to the extent of forming macroscopic pseudo-tumor. Otherwise, previous study revised that FDG PET/CT showed no substantial advantage compared to routine radiology in detecting pancreas, bile duct and retroperitoneum [28]. It might be due to the low background uptake of FAPI in theses organs compared with FDG.

In line with previous reports [17, 24, 26], lymph node involvement was FAPI-negative in our study because most IgG4-RD lymphadenopathy is due to the infiltration of plasma cells rather than fibrosis [29]. IgG4-RD lymphadenopathy is indistinguishable from reactive and neoplastic lymph nodes in conventional scanning [30], which can lead to an over- or underestimation of disease burden. Also, the biopsies from lymph nodes are not acceptable for use in weighting the immunostaining domain for classification [1]. The difference of FDG and FAPI uptake in lymph nodes may help to avoid the misleading biopsy site of extensive lymphadenopathy detected by FDG.

Several studies had reported that total MTV and total TLG of FDG PET/CT were significantly correlated with serum biomarkers in IgG4-RD [11, 13, 31]. In the present study, positive correlations between TL-FAPI and the levels of ESR, IgG4 and IgG4-RI was observed, whereas SUV_max_ was not. After regression analysis, the TL-FAPI was identified as an effective indicator of IgG4-RD activity. Schmidkonz C et al. reported that ^68^Ga-FAPI-04 tracer uptake was associated with fibrotic but not inflammatory lesions of IgG4-RD [16]. Among the pathophysiology of periprosthetic joint infection and aseptic loosening, FAPI uptake was low at the lesion site in the early stage of systemic inflammation and showed no correlation with CRP and IL-6 [32]. In our study, TL-FAPI also revealed negative correlations with CRP and IL-6, while TL-FAPI was positively correlated with ESR. It might due to the hypergammaglobulinemia in IgG4-RD leading to the increased ESR [33], as TL-FAPI indeed reflected the disease activity of IgG4-RD.

There are still some limitations in this study. The number of patients was small, and the longitudinal analysis was not fully performed. To demonstrate equal performance between ^18^F-FAPI PET/CT and ^68^Ga-FAPI PET/CT, a head-to-head comparison of these two radiotracers should have been performed. Meanwhile, only three patients had acceptable immunostaining parameters making it impossible to evaluate the relationship between histopathological findings and PET/CT findings. Meanwhile, we did not correlate FAPI uptake with fibrotic level due to the lack of reliable biomarkers. Also, the correlation between TL-FAPI and disease activity was not very strong. A model consisting of clinical manifestations, laboratory biomarkers, and imaging parameters was required for evaluating the disease activity of IgG4-RD. This study was a pilot study at a single center, and more high evidence studies are needed to verify the reliability of the PET/CT parameters in the assessment of disease activity in IgG4-RD.

## Conclusions

Our results underlined the potential value of [^18^F]AlF-NOTA-FAPI-04 in the evaluation of IgG4-RD, and it might have advantage in detecting some lesions, such as pancreas and lacrimal gland. These positive findings highlighted that the TL-FAPI could be an effective and easy-to-use imaging indicator of disease activity in IgG4-RD.

## Data Availability

All data generated or analyzed during this study are included in this published article and its supplementary information files.
